# Issues in Optical Diffraction Theory

**DOI:** 10.6028/jres.114.007

**Published:** 2009-04-01

**Authors:** Klaus D. Mielenz

**Affiliations:** National Institute of Standards and Technology (ret.) Gaithersburg, MD 20899-8440

**Keywords:** bidirectional scalar diffraction, continuously differentiable field components, Fresnel, Kirchhoff, near-field, partial coherence, plane apertures, polarization, pseudo-vectorial theories, Rayleigh, Sommerfeld, transmission coefficients

## Abstract

This paper focuses on unresolved or poorly documented issues pertaining to Fresnel’s scalar diffraction theory and its modifications. In Sec. 2 it is pointed out that all thermal sources used in practice are finite in size and errors can result from insufficient coherence of the optical field. A quarter-wave criterion is applied to show how such errors can be avoided by placing the source at a large distance from the aperture plane, and it is found that in many cases it may be necessary to use collimated light as on the source side of a Fraunhofer experiment. If these precautions are not taken the theory of partial coherence may have to be used for the computations.

In Sec. 3 it is recalled that for near-zone computations the Kirchhoff or Rayleigh-Sommerfeld integrals are applicable, but fail to correctly describe the energy flux across the aperture plane because they are not continuously differentiable with respect to the assumed geometrical field on the source side. This is remedied by formulating an improved theory in which the field on either side of a semi-reflecting screen is expressed as the superposition of mutually incoherent components which propagate in the opposite directions of the incident and reflected light.

These components are defined as linear combinations of the Rayleigh-Sommerfeld integrals, so that they are rigorous solutions of the wave equation as well as continuously differentiable in the aperture plane. Algorithms for using the new theory for computing the diffraction patterns of circular apertures and slits at arbitrary distances *z* from either side of the aperture (down to *z* = ± 0.0003 *λ*) are presented, and numerical examples of the results are given. These results show that the incident geometrical field is modulated by diffraction before it reaches the aperture plane while the reflected field is spilled into the dark space. At distances from the aperture which are large compared to the wavelength *λ* these field expressions are reduced to the usual ones specified by Fresnel’s theory. In the specific case of a diffracting half plane the numerical results obtained were practically the same as those given by Sommerfeld’s rigorous theory.

The modified theory developed in this paper is based on the explicit assumption that the scalar theory of light cannot explain plolarization effects. This premise is justified in Sec. 4, where it is shown that previous attempts to do so have produced dubious results.

## 1. Introduction

**Fig. 1:**
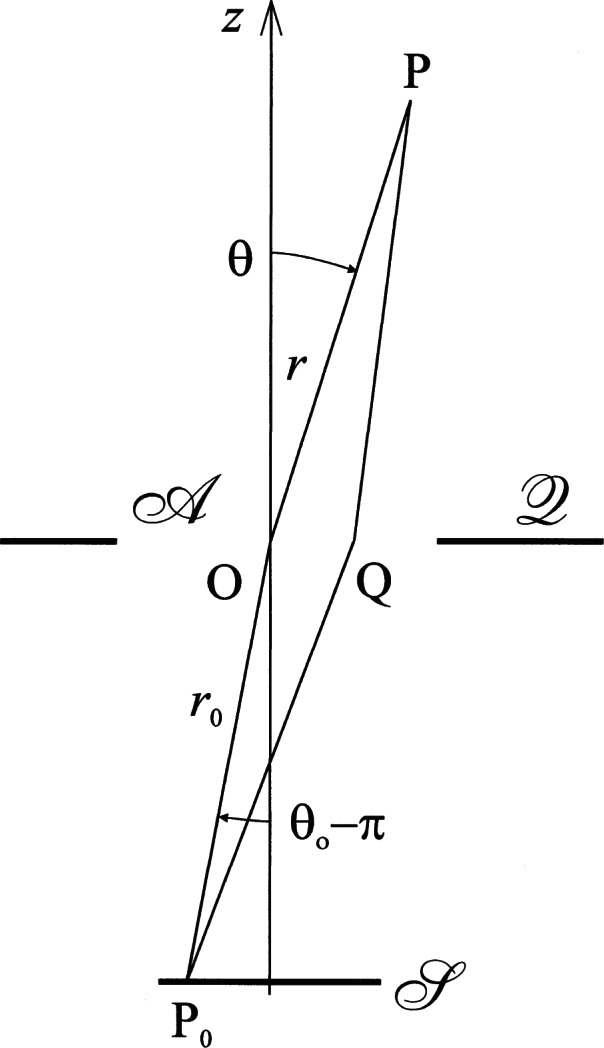
Geometry pertaining to Fresnel’s diffraction integral.

Calculations pertaining to the diffraction of light by an aperture are typically based on geometric assumptions as illustrated in Fig. 1. A plane screen Q containing an aperture A of width 2*w* is illuminated by a quasi-monochromatic source S of width 2*s* and circular wave number *k* = 2π/*λ*, P_0_ is a source point, Q is a point inside the aperture, and P is the point at which diffraction is observed. A point inside the aperture (usually the center) is chosen as the origin O, and cartesian or polar coordinates are used so that

$$\begin{array}{l} P_{0}=\left(x_{0},y_{0},z_{0}\right)=r_{0}\left(\cos\phi_{0}\sin\theta_{0},\sin\phi_{0}\sin\theta_{0},\cos\theta_{0}\right),\\ Q=\left(\xi ,\eta ,0\right)=q\left(\cos\chi ,\sin\chi ,0\right),\\ P=\left(x,y,z\right)=r\left(\cos\phi \sin\theta ,\sin\phi \sin\theta ,\cos\theta \right). \end{array}$$ (1)

The first mathematical theory of diffraction was derived in Fresnel’s 1819 *Memoir on the Diffraction of Light* [1] from a surprisingly minimal set of assumptions:

Monochromatic light is a harmonic wave motion that can be described in terms of a scalar wave function, *U*(P)exp(− i*ω t*), where |*U*(P)|^2^ is the irradiance at P, *ω* = 1/*kc*, is the circular frequency *k* = 2π/*λ*, is the circular wave number and *c* is the speed of light.
The summation of Huygens’ wavelets is best carried out on a wave front that coincides with the aperture; otherwise the computations will be too complicated.
A plane aperture is assumed, which implies that the source must be distant as well as paraxial *(r*_0_ > 2*w*^2^/*λ*, cos *θ*_0_ ~ 0),^1^ so that the incident wave front can, within acceptable tolerances, be assumed to lie inside the aperture.
The field on the source side of the screen is not affected by the presence of the screen (St. Venant’s hypothesis).^2^ For an infinitesimally small source with radiant intensity *I* which is located at the point P_0_ in Fig. 1, this leads to *U*(Q) = √*Ī* exp (i*kP_0_Q*)*/P_0_Q.*
The Huygens’ wavelets originating at Q are anisotropic spherical waves that oscillate a quarter period ahead of the incident field and whose amplitudes are scaled by the factor 1/*λ*. As the corresponding effect at the point of observation P is attributable to the central Fresnel zone at Q acting alone, there is no need to know the nature of this anisotropy and hence it follows that d*U* (P) = (i/*λ*)*U*(Q) exp (i*kQP*)*/QP.*
On account of the denominator *λ QP* in the last expression it is necessary to assume that *QP* is a large distance (*r* ≫ *λ*), and that P must also be paraxial (cos *θ* ≈ 1) as the direction of observation cannot be substantially different from the direction of incidence.

Combining these expression, Fresnel found

$$\begin{array}{l} U_{F}\left(P\right)=\frac{i\sqrt{I}}{\lambda }\int_{A}\mathrm{dQ}\frac{\exp\left[ik\left(P_{0}Q+QP\right)\right]}{P_{0}Q\;QP}\\ ∼\frac{i\sqrt{E_{\mathrm{geom}}}\exp\left[ik\left(r_{0}+r\right)\right]}{\lambda r}\int_{A}dQ\exp\left[ik\Delta \left(Q\right)\right],\;z\gg \lambda , \end{array}$$ (2)

where $E_{\mathrm{grom}}=I/r_{0}^{2}$ is the incident geometrical irradiance of the aperture plane and Δ(*Q*) is the path difference (*P*_0_*Q* + *QP*) – (*r*_0_ + *r*).

Although a relic of the early 19^th^ century, the Fresnel diffraction integral (2) is still used today in its original form and has remained a most useful, reliable tool for diffraction calculations that have consistently yielded results which agree with experience and are well documented [2]. Accordingly, it is commonly regarded as a cornerstone of diffraction theory and its validity for midfield applications remains unchallenged. On the other hand, the classical theory of optical diffraction is limited in scope as well as physical significance by several issues which are still unresolved, misunderstood, or poorly documented. For example:

The assumption of an infinitesimally small isotropic point source is seldom justified. All thermal sources are finite in size, so that errors may arise from an insufficient coherence of the optical field. As shown in Sec. 2, below, this can be avoided by invoking the theory of partial coherence, or by negating the error in the first place by designing a diffraction experiment so that the aperture illumination is “almost” coherent.
As Fresnel’s integral is not applicable for computations in the near zone, it would seem that in this case the more accurate Rayleigh-Sommerfeld or Kirchhoff boundary-value integrals [Eqs. (5a–c), below] can be used with confidence. This is, however, not the case as these integrals do not correctly describe the field in the proximity of the aperture screen. In Sec. 3, this problem is solved by constructing an improved theory in which St. Venant’s hypothesis is abandoned and the field on both sides of the screen is expressed in terms of linear combinations of the Rayleigh-Sommerfeld integrals.
The standard solutions of the Fresnel diffraction integral (2) for circular apertures and apertures bounded by straight edges are models of mathematical elegance, but notoriously difficult to evaluate in practice. The numerical methods used in this paper not as elegant, but easier to use. As they are rigorously correct, they can be used at arbitrary distances from the screen, and this is recommended.
Initially, Fresnel believed that light is akin to longitudinal sound waves but prior to the completion of his theory he discovered, in collaboration with Arago,^3^ that it is a transverse wave. He pondered the obvious question how this discovery affected the theory of diffraction and concluded that “*the arguments and computations contained in the Memoir harmonize quite as well with this new hypothesis as with the preceding, because they are independent of the actual directions of the vibrations.*” In other words, the scalar approach makes it impossible to describe the diffraction of polarized light. This is confirmed in Sec. 4, below.

## 2. Coherence Issues

Extended thermal sources employed in diffraction experiments are used either by themselves or followed by a limiting aperture. In either case, the diffraction pattern due to the principal aperture A can be evaluated using a generalized Fresnel integral derived by this author from the general equations for the propagation of cross-spectral density in a partially coherent optical field [3]. The case of an incoherent source used by itself is a straightforward generalization of Eq. (2) and leads to the following expression for the irradiance at the point P in Fig. 1,

$$E\left(P\right)=\frac{1}{{\left(\lambda r\right)}^{2}}\int_{S}{\mathrm{dP}}_{0}L\left(P_{0}\right){\left|\int_{A}\mathrm{dQ}\exp\left[ik\left(P_{0}Q+QP\right)\right]\right|}^{2},$$ (3a)

where *L*(P_0_) is the source radiance. In the case of a thermal source followed by an aperture located in the plane S, it is necessary to use the van Cittert-Zernike theorem to calculate the cross-spectral density *W*(*P*_0_, *P*_0_,*ω*) on S and then find E(P) from the expression

$$\begin{array}{l} E\left(P\right)=\frac{1}{\lambda^{4}r^{2}}\int_{S}{\mathrm{dP}}_{0}{dP^{\prime }}_{0}\frac{W\left(P_{0},P_{0}^{\prime }\omega \right)}{P_{0}QP_{0}Q^{\prime }}\\ \;\int_{A}\mathrm{dQ}dQ^{\prime }\exp\left[ik\left(P_{0}Q+QP-P_{0}^{\prime }Q^{\prime }+Q^{\prime }P\right)\right], \end{array}$$ (3b)

where P_0_ and P′_0_ are two points on S, Q and Q′ are points in A, and *ω* is the circular frequency of the light. These equations were applied in Ref. [4] to the specific case of concentric circular apertures and yielded complicated but closed solutions in terms of Lommel functions.

The use of Eqs. (3a) and (3b) for practical computations is a very tedious task. Therefore, it may be desirable to avoid the need for these computations in the first place by designing diffraction experiments so that the aperture illumination will be “almost” coherent and the Fresnel integral (2) can still be used, in spite of the finite size of a given source. This can be done as follows, assuming the worst case of a totally incoherent source as implied by Eq. (3a), considering a concentric arrangement of source and aperture as in Fig. 1, and then applying the quarter-wave criterion so that the path lengths *P*_0_ *Q* do not vary by more than *λ*/4. According to Fig. 1, the extreme values of *P*_0_*Q are r*_0_ and $\sqrt{r_{0}^{2}+{\left(s+w\right)}^{2}}\approx r_{0}+{\left(s+w\right)}^{2}/2r_{0}$, and thus the desired criterion is (*s*+*w*)^2^/*r*_0_ ≤ λ/2 or

$$r_{0}\geq \frac{2{\left(s+w\right)}^{2}}{\lambda }.$$ (4a)

As shown in Table 1, the corresponding minimum distances *r*_0,min_ vary significantly with the sizes of source and aperture, ranging from a few millimeters for small sources and apertures to hundreds of meters for large ones. In the latter case Eq. (4a) can only be satisfied by placing the source in the focal plane of a collimator lens, as on the source side of a Fraunhofer diffraction experiment.^4^

The coherence criterion (4a) assures a coherent and uniform aperture field so that the path lengths (*P*_0_*Q* + *QP*) are all effectively equal to (*r*_0_ + *QP*), as if the incident field is a plane wave propagating in the direction of the *z*-axis and $U_{\mathrm{geom}}=\sqrt{E_{\mathrm{geom}}}\exp\left(i\;kz\right)$. Under these conditions Eq. (3a) is reduced to a product of independent integrals over source and aperture, and it is easy to show that the first of these, $\int_{S}{\mathrm{dP}}_{0}L\left(P_{0}\right)$ is equal to the geometrical irradiance of the aperture plane if Lambert’s law is assumed. That is,

$$\begin{array}{l} E\left(P\right)=\frac{1}{{\left(\lambda r\right)}^{2}}\int_{S}{\mathrm{dP}}_{0}L\left(P_{0}\right)\times |\int_{A}\mathrm{dQ}\exp\left[ik\left(r_{0}+QP\right)\right]\\ \;=\frac{E_{\mathrm{geom}}}{{\left(\lambda r\right)}^{2}}{\left|\int_{A}\mathrm{dQ}\exp\left(ikQP\right)\right|}^{2}, \end{array}$$ (4b)

which is the squared modulus of Eq. (2) for *P*_0_*Q* = *r*_0_.

## 3. Rigorous Theory

### 3.1 Background

Fresnel was aware that the spherical wavelets assumed in his derivation of Eq. (2) cannot be isotropic, because otherwise light would also travel back toward the source. To avoid this contradiction, so-called inclination factors can be introduced to assure that the amplitudes of the wavelets are zero in the reverse direction. As mentioned earlier, Fresnel did not know the form of these factors and simply omitted them, assuming correctly that they are not needed in a paraxial theory. The question of inclination factors was answered by the publication of Helmholtz’ theorem in 1859 [5].^5^ Whereas Fresnel had only stipulated that the screen Q in Fig. 1 must be large enough to prevent the leakage of light around its edges, Helmholtz imagined it to be an infinitely large, closed surface which does not contain the primary source, and then invoked Green’s formula to express the diffracted field *U*(P) as a surface integral of the form prescribed by Fresnel’s first assumption. This provided the missing inclination factors in the form of the normal derivatives ∂*U*(Q)/∂*n*, which vanish when the light propagates in the direction tangential to the screen. In short, Helmholtz’ theorem states that the diffracted field is confined to the inside of the surface S but null on the outside, and thus it merges Huygens’ principle and St. Venant’s hypothesis into one.

The principal solutions of Helmholtz’ theorem are the familiar Rayleigh-Sommerfeld and Kirchhoff diffraction integrals [6,7,8],

$$\begin{array}{l} U_{\mathrm{RS}}^{\left(p\right)}\left(P\right)=-\frac{1}{2\pi }\int_{A}\mathrm{dQ}\frac{\partial U_{\mathrm{geom}}\left(Q\right)}{\partial z}\frac{\exp\left(ikQP\right)}{QP}\\ \;=-\frac{ik\sqrt{E_{\mathrm{geom}}}}{2\pi }\int_{A}\mathrm{dQ}\frac{\exp\left(ikQP\right)}{QP},\;z\geq 0, \end{array}$$ (5a)

$$\begin{array}{l} U_{\mathrm{RS}}^{\left(s\right)}\left(P\right)=\frac{1}{2\pi }\int_{A}\mathrm{dQ}\;U_{\mathrm{geom}}\left(Q\right)\frac{\partial }{\partial z}\left(\frac{\exp\left(ikQP\right)}{QP}\right)\\ \;=\frac{1}{ik}\frac{\partial U_{\mathrm{RS}}^{\left(p\right)}}{\partial z},\;z\geq 0, \end{array}$$ (5b)

$$U_{K}\left(P\right)=\frac{1}{2}\left[U_{\mathrm{RS}}^{\left(p\right)}\left(P\right)+U_{\mathrm{RS}}^{\left(s\right)}\left(P\right)\right],$$ (5c)

where the right-most expressions in (5a,b) were obtained by assuming that the coherence criterion (4a) is satisfied so that $\sqrt{E_{\mathrm{grom}}}\exp\left(i\;kz\right)$ could be substituted for *U*_geom_ (Q). It is well known and easy to show that in the mid zone these expressions are all reduced to the Fresnel integral (2) so that

$$U_{\mathrm{RS}}^{\left(p\right)}\left(P\right)=U_{\mathrm{RS}}^{\left(s\right)}\left(P\right)=U_{K}\left(P\right)=U_{F}\left(P\right),\;z\gg \lambda .$$ (5d)

It is equally well known that the Rayleigh-Sommerfeld integrals reproduce the boundary values assumed in their derivation,

$$\begin{array}{l} \frac{\partial U_{\mathrm{RS}}^{\left(p\right)}\left(Q\right)}{\partial z}\equiv ik\sqrt{E_{\mathrm{geom}}},\\ U_{\mathrm{RS}}^{\left(s\right)}\left(Q\right)\equiv \sqrt{E_{\mathrm{geom}}},Q\in A, \end{array}$$ (5e)

but not the corresponding values, $U_{\mathrm{RS}}^{\left(p\right)}\left(Q\right)=\sqrt{E_{\mathrm{geom}}}$ and $\partial U_{\mathrm{RS}}^{\left(s\right)}\left(Q\right)/\partial z=ik\sqrt{K_{\mathrm{geom}}}$. Therefore, the wave functions defined by Eqs. (5a–c) are different from one another in the near zone and not continuously differentiable in the aperture, so that none expresses the diffracted field as an analytical continuation of the assumed geometrical field on the source side.

In order to overcome this failure of the classical boundary-value theories to describe a smooth flow of energy across the aperture plane, so-called “rigorous” theories have been formulated in which St. Venant’s hypothesis is abandoned and it is assumed that the incident geometrical is modified by diffraction before it reaches the screen. Hence, its values on the source side can be determined by postulating that the overall field is continuously differentiable inside the aperture. The most important, and by far most successful treatment of this type is Sommerfeld’s rigorous theory of diffraction by a half plane [7, 9], which is expressed in closed form and involves no approximations of any sort. Other examples are the Rayleigh-Bouwkamp [6, 10] and Levine-Schwinger [11] theories, which were intended to define the transmission coefficients of very small apertures but are approximations based on assumed aperture field distributions expressed as a series of algebraic functions. Undoubtedly, the rigorous treatment of diffraction problems is more powerful than the boundary-value approach but unfortunately some of the most common problems, such as diffraction by circular apertures or slits of arbitrary sizes, have so far not been solved rigorously.

The reasons for this deficiency of the prior literature were analyzed in a recent study [12] which included a numerical comparison of the respective half-plane results obtained from the Sommerfeld and Rayleigh-Sommerfeld theories. The theory derived in the following subsection is based on the findings of this study, and it is important that it involves concepts not found in other theories:

The frequently encountered association of the Rayleigh-Sommerfeld integrals with parallel or perpendicularly polarized light is abandoned and nothing is assumed about them, except that they are independent solutions of the wave equation which satisfy the respective boundary conditions specified in Eq. (5e), above.
Similarly, the customary distinction between “black” and “metallic” screens is abandoned. The physical nature of the screen is not specified, except that it has a certain reflectance |*ρ*|^2^ which can be used as a scale factor to describe different types of screens.
In the general case of a partially reflecting screen there will be two mutually incoherent diffraction patterns, one in the forward direction of the incident light and the other in the reverse direction of the reflected light. The latter is usually not observable, but must be taken into account because its presence affects the radiant flux transmitted in the forward direction.

### 3.2 Derivations

Consider the diffraction of light by a plane aperture A in an infinite, infinitesimally thin screen Q which is illuminated by a source S, as shown in Fig. 1. Assume that Q is a specular reflector with amplitude reflectance *ρ* and that the coherence condition (4a) is satisfied, so that the incident geometric field and its reflection can be expressed in the form of plane waves, $U_{\mathrm{geom}}=\sqrt{E_{\mathrm{geom}}}\exp\left(ikz\right)$ and ${\hat{U}}_{\mathrm{geom}}=\rho \sqrt{E_{\mathrm{geom}}}\exp\left(\text{-}ikz\right)$. Under these conditions, the diffracted field will likewise be composed of mutually incoherent components *U*(P) and $\hat{U}\left(P\right)$, which travel in opposite directions. The task at hand is to define these components so that each is continuously differentiable inside the aperture. Using a normalized notation so that $U\left(P\right)=\sqrt{E_{\mathrm{geom}}}u\left(P\right)$ and $\hat{U}\left(P\right)=\sqrt{E_{\mathrm{geom}}}\hat{u}\left(P\right)$, we express *u* (P) and û(P) in the form of the trial solutions,

$$\begin{array}{l} u\left(P\right)=\{\begin{array}{c} u_{+}\left(P\right),\;z>0\\ \exp\left(ikz\right)+u_{-}\left(P\right),\;z<0 \end{array}\\ \hat{u}\left(P\right)=\rho \{\begin{array}{c} {\hat{u}}_{+}\left(P\right),\;z>0\\ {\hat{u}}_{-}\left(P\right),\;z<0 \end{array} \end{array}$$ (6a)

where *u* ± (P) and *û* ± (P) are initially unknown field components which are attributed to diffraction effects.

As these unknown quantities must have certain prerequisite properties such as obeying the wave equation as well as the infinity and edge conditions [7, 13], and because the Rayleigh-Sommerfeld integrals (5a, b) have these properties, *u*_±_ (P) and *û*_±_ (P) can be defined as linear combinations of the form

$$\begin{array}{l} u_{\pm }\left(P\right)=au_{\mathrm{RS}}^{\left(p\right)}\left(P\right)\pm bu_{\mathrm{RS}}^{\left(s\right)}\left(P\right),\\ {\hat{u}}_{\pm }\left(P\right)=\hat{a}u_{RS}^{\left(p\right)}\left(\hat{P}\right)\pm \hat{b}u_{\mathrm{RS}}^{\left(s\right)}\left(\hat{P}\right), \end{array}$$ (6b)

where $\hat{P}=\left(x,y,-z\right)$ is the reflection of the point of observation P, defined so that $u_{\mathrm{RS}}^{\left(p,s\right)}\left(\hat{P}\right)$ is a valid expression when *z* < 0.

The coefficients *a*, *b* and $\hat{a}$, $\hat{b}$in (6b) can now be determined by postulating that the forward field is equal to Fresnel’s integral *u*_F_ (P) in the positive mid zone and equal to the incident geometrical field in the negative mid zone. The respective values for the reverse field are assumed to be zero and $U_{F}\left(\hat{P}\right)$. That is,

$$u\left(P\right)=\{\begin{array}{c} \left(a+b\right)u_{F}\left(P\right)=u_{F}\left(P\right),\;z\gg \lambda \\ \exp\left(ikz\right)+\left(a-b\right)u_{F}\left(P\right)=\exp\left(ikz\right),\;-z\gg \lambda \end{array}$$ (6c)

$$\hat{u}\left(P\right)=\rho \{\begin{array}{c} \left(\hat{a}+\hat{b}\right)u_{F}\left(\hat{P}\right)=0,\;z\gg \lambda \\ \left(\hat{a}-\hat{b}\right)u_{F}\left(\hat{P}\right)=u_{F}\left(\hat{P}\right),\;-z\gg \lambda \end{array}$$ (6d)

where Eq. (5d) was used to let $u_{\mathrm{RS}}^{\left(p\right)}=u_{\mathrm{RS}}^{\left(s\right)}=u_{F}$ when z/λ is large.

Equations (6c,d) are satisfied if $a=b=\frac{1}{2}$, $\hat{a}=-\hat{b}=\frac{1}{2}$, so that

$$\begin{array}{l} u\left(P\right)=\{\begin{array}{c} u_{K}\left(P\right),\;z>0\\ \exp\left(ikz\right)+{\hat{u}}_{K}\left(\hat{P}\right),\;z<0 \end{array}\\ \hat{u}\left(P\right)=\rho \{\begin{array}{c} {\hat{u}}_{K}\left(P\right),\;z>0\\ u_{K}\left(\hat{P}\right),\;z<0 \end{array} \end{array}$$ (6e)

where

$$\begin{array}{l} u_{K}\left(P\right)=\frac{1}{2}\left[u_{\mathrm{RS}}^{\left(p\right)}\left(P\right)+u_{\mathrm{RS}}^{\left(s\right)}\left(P\right)\right],\\ {\hat{u}}_{K}\left(P\right)=\frac{1}{2}\left[u_{\mathrm{RS}}^{\left(p\right)}\left(P\right)-u_{\mathrm{RS}}^{\left(s\right)}(P)\right]. \end{array}$$ (6f)

It remains to show that these expressions are continuously differentiable inside the aperture. In the case of *u*(P), this will be the case if

$$\begin{array}{l} \frac{1}{2}\left[u_{\mathrm{RS}}^{\left(p\right)}\left(Q\right)+u_{\mathrm{RS}}^{\left(p\right)}\left(Q\right)\right]=\\ \;1+\frac{1}{2}\left[u_{\mathrm{RS}}^{\left(p\right)}\left(Q\right)-u_{\mathrm{RS}}^{\left(p\right)}\left(Q\right)\right], \end{array}$$ (6g)

$$\begin{array}{l} \frac{1}{2}\frac{\partial }{\partial z}\left[u_{\mathrm{RS}}^{\left(p\right)}\left(Q\right)+u_{\mathrm{RS}}^{\left(p\right)}\left(Q\right)\right]=\\ \;ik+\frac{1}{2}\frac{\partial }{\partial \left(-z\right)}\left[u_{\mathrm{RS}}^{\left(p\right)}\left(Q\right)-u_{\mathrm{RS}}^{\left(p\right)}\left(Q\right)\right], \end{array}$$ (6h)

which according to Eq. (5e) is true. This argument can be repeated to prove that, likewise, *û*(P) is continuously differentiable when P = Q.

Equations (6e) represent the main result of this Section. The corresponding forward and reverse irradiances of the field are

$$E\left(P\right)/E_{\mathrm{geom}}={\left|u\left(P\right)\right|}^{2}=\{\begin{array}{c} {\left|u_{K}\left(P\right)\right|}^{2},\;z>0\\ {\left|\exp\left(ikz\right)+{\hat{u}}_{K}\left(\hat{P}\right)\right|}^{2},\;z<0 \end{array}$$ (6i)

$$\hat{E}\left(P\right)/E_{\mathrm{geom}}={\left|\hat{u}\left(P\right)\right|}^{2}=\{\begin{array}{c} {\left|{\hat{u}}_{K}\left(P\right)\right|}^{2},\;z>0\\ {\left|u_{K}\left(\hat{P}\right)\right|}^{2},\;z<0 \end{array}$$ (6j)

Like *u*(P) and *û*(P) themselves, these expressions are continuously differentiable inside the aperture and thus imply a smooth, bidirectional flow of energy from one side to the other. It should be noted that, in these expressions, the roles of Kirchhoff’s integral *u*_K_(P) and its counterpart *û*_K_(P) are reversed on opposite sides of the screen, so that they counterbalance each other and the discontinuities of the Rayleigh-Sommerfeld integrals $u_{\mathrm{RS}}^{\left(p,s\right)}$ are eliminated.

The general properties of the diffracted field defined by these quantities can be inferred from the above-mentioned fact that the differences between the Rayleigh-Sommerfeld integrals are pronounced only in the immediate proximity of the screen and vanish in the mid zone. Thus the forward component ${\hat{u}}_{K}\left(\hat{P}\right)$ in the lower Eq. (6i) disappears as the Fresnel limit is approached, and the reverse component *û*_K_(P) in the upper equation (6j) is an evanescent wave which also vanishes in the Fresnel limit. For |*z*| ≫*λ*, the forward field is the same as in Fresnel’s theory and the reverse field is a mirror image of the forward Fresnel pattern.

### 3.3 Implementation

#### 3.3.1 General

The formulae of the preceding section are unsuited for numerical computations unless they can be reduced to single integrals that can be evaluated without simplifying assumptions which might degrade their accuracy. Fortunately, the rare instances in which this can be done include two cases of great practical importance: circular apertures and apertures bounded by straight edges when illuminated by normally incident light. The single integrals obtained in these two cases can readily be evaluated by numerical methods based on the algorithms described in Sects. 3.3.2 and 3.3.3, below. The use of these algorithms on a personal computer with standard spreadsheet software is straightforward and yields numerical results that were found to be everywhere finite except at distances |*z*| <0.01*λ* from the aperture plane.

The same algorithms can also used at distances *z* ≫*λ*, and this is recommended because the standard analytical solutions of Fresnel’s integral (2) are notoriously difficult to use on a personal computer. For example, the algorithm defined by Eqs. (8a–c), below, is less tedious than implementing Lommel’s analytical solution of the Fresnel diffraction pattern of circular apertures in terms of infinite series of Bessel functions [2]. Likewise, the use of Eqs. (11a,b) is easier than the computation of complex Fresnel integrals by Taylor series and polynomial approximations [14]. The algorithms (8a–c) and (11a,b) have the added advantage of being rigorously accurate, and it should also be noted that in most cases it is not necessary to compute the reverse field. On the other hand, the methods described in this paper can only be used for normally incident light. It should also be mentioned that, owing to the highly structured nature of the diffraction patterns, the use of any of these methods can be cumbersome when wide apertures are considered.

#### 3.3.2 Circular Apertures

##### 3.3.2.1 Algorithms

**Fig. 2:**
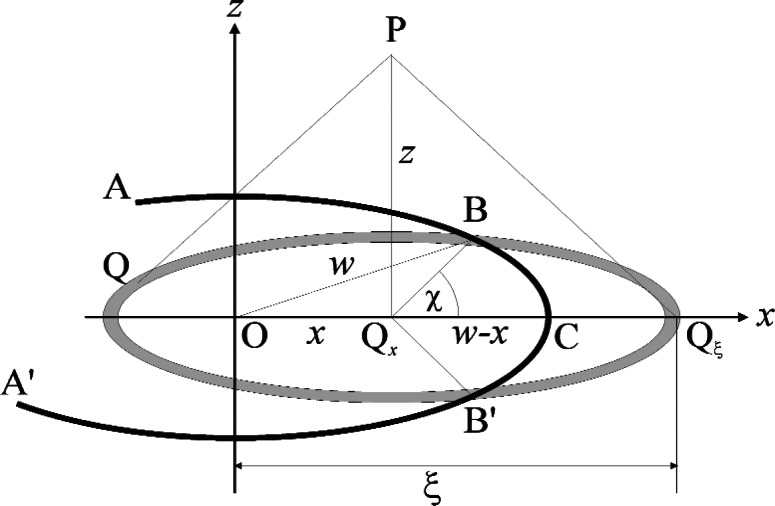
Notation used for circular apertures.

Let *ABCB*′*A*′ be the rim of a circular aperture of radius *w* which is illuminated by normally incident coherent light and is centered on the coordinate origin, as shown in Fig. 2. As the corresponding diffraction pattern must be rotationally symmetrical about the *z*-axis it will be sufficient to consider its variation in the *xz*-plane, and thus the point of observation is chosen as P = (*x*, 0, *z*). The integrals (5a,b) may then be reduced to single integrals by defining the area elements dQ so that they are all concentric with the projection Q_0_ = (*x*, 0, 0) of P onto the aperture plane and coincide with the circles *QBQ_ξ_B*′ shown in the figure, where Q*_ξ_* = (*ξ*, 0, 0) is the right-most point at which these circles intersect the *x*-axis. Under these conditions the phases *kQP* will be constant and equal to

$$\begin{array}{l} \beta \equiv k\;QP=k\;Q_{\xi }P=k\sqrt{{\left(\xi -x\right)}^{2}+z^{2}}=\sqrt{v^{2}+{\left(kz\right)}^{2}},\\ v=k\left(\xi -x\right) \end{array}$$ (7a)

everywhere on these area elements and the integration can be carried out over *ξ* – *x* alone. As also indicated in the figure, these area elements are in general not fully contained in the aperture and must therefore be evaluated as

$$\begin{array}{l} \mathrm{dQ}=2\pi d\left(\xi -x\right)\left(\xi -x\right)\left(1-\chi /\pi \right)\\ =\left(2\pi /k^{2}\right)dv\;v\left(1-\chi /\pi \right), \end{array}$$ (7b)

where 2*χ* is the angle subtended by the obstructed arc *BQ_ξ_ B*′ and is given by

$$\begin{array}{l} \cos\chi =\frac{w^{2}-x^{2}-{\left(\xi -x\right)}^{2}}{2x\left(\xi -x\right)}\\ \;=\frac{{\left(kx\right)}^{2}-{\left(kx\right)}^{2}-v^{2}}{2kxv}, \end{array}$$ (7c)

or *η* = 0 or π, as appropriate, when the right-hand side of (7c) exceeds ± 1. Hence one finds, using Eqs. (5a,b) and (7a,b),

$$\begin{array}{l} u_{\mathrm{RS}}^{\left(p\right)}\left(x,z\right)=-\frac{ik^{2}}{2\pi }\int \mathrm{dQ}\frac{\exp\left(i\beta \right)}{\beta }\\ \;=-i\int dvv\left(1-\chi /\pi \right)\frac{\exp\left(i\beta \right)}{\beta },z>0, \end{array}$$ (7d)

$$\begin{array}{l} u_{\mathrm{RS}}^{\left(s\right)}\left(x,z\right)=\frac{1}{ik}\frac{\partial u_{\mathrm{RS}}^{\left(s\right)}\left(x,z\right)}{\partial z}\\ \;=-kz\int dvv\left(1-\chi /\pi \right)\left(i-\frac{1}{\beta }\right)\frac{\exp\left(i\beta \right)}{\beta },\\ z>0, \end{array}$$ (7e)

so that the integrals defined in Eq. (6e) are now given by

$$\begin{array}{l} u_{K}\left(x,z\right)=\int dvv\left(1-\chi /\pi \right)C\left(\beta \right),\;z>0,\\ C\left(\beta \right)=\left[\left(\frac{1}{\beta }+\frac{kz}{\beta^{2}}\right)\sin\beta +\frac{kz}{\beta^{3}}\cos\beta \right]\\ \;-i\left[\left(\frac{1}{\beta }+\frac{kz}{\beta^{2}}\right)\cos\beta -\frac{kz}{\beta^{3}}\sin\beta \right], \end{array}$$ (7f)

$$\begin{array}{l} {\hat{u}}_{K}\left(x,z\right)=\int dvv\left(1-\chi /\pi \right)\hat{C}\left(\beta \right),\;z>0,\\ \hat{C}\left(\beta \right)=\left[\left(\frac{1}{\beta }-\frac{kz}{\beta^{2}}\right)\sin\beta -\frac{kz}{\beta^{3}}\cos\beta \right]\\ -i\left[\left(\frac{1}{\beta }-\frac{kz}{\beta^{2}}\right)\cos\beta +\frac{kz}{\beta^{3}}\sin\beta \right]. \end{array}$$ (7g)

The limits of these integrals are *v* = 0 to *k*(*w* + *x*) when *x* ≤ *w*, and *v* = *k* (*x* – *w*) to *k*(*x* + *w*) when *x* ≥ *w*. In the first of these ranges it is assumed that *χ* ≡ 0 when *v* ≤ *k*(*x* – *w*).

In order to evaluate these integrals numerically, divide the aperture radius *w* into *N* equal elements, and let *ξ* – *x* = *nw* / *N*, *x* = *mw* / *N*. Hence, d*v* = *kw* / *N*, *v* = *nkw*/*N*, and therefore

$$\begin{array}{l} u_{K}\left(x,z\right)=u_{K}\left(m,z\right)={\left(\frac{kw}{N}\right)}^{2}\sum_{n}\left(n-\frac{1}{2}\right)\left(1-\chi_{n,m}\right)C\left(\beta_{n}\right),\\ z>0, \end{array}$$ (8a)

$$\begin{array}{l} {\hat{u}}_{K}\left(x,z\right)={\hat{u}}_{K}\left(m,z\right)={\left(\frac{kw}{N}\right)}^{2}\sum_{n}\left(n-\frac{1}{2}\right)\left(1-\chi_{n,m}\right)\hat{C}\left(\beta_{n}\right),\\ z>0, \end{array}$$ (8b)

where

$$\begin{array}{l} \chi_{n,m}=0\;\mathrm{if}\;n\leq m,\;\chi_{n,m}=\cos^{-1}\frac{N^{2}-m^{2}-{\left(n-\frac{1}{2}\right)}^{2}}{2m\left(n-\frac{1}{2}\right)}\\ \mathrm{if}\;n>m, \end{array}$$ (8c)

$$\beta_{n}=\sqrt{\frac{{\left(n-\frac{1}{2}\right)}^{2}{\left(kw\right)}^{2}}{N^{2}}}+{\left(kz\right)}^{2},$$ (8d)

all quantities being evaluated at the mid-points of the summation elements. The ranges of summation in Eqs. (8a) are 1 ≤ *n* ≤ m + *N* if *n* ≤ *m* and *m* − *N* < *n* ≤ *m* + *N*. The number *N* of summation elements used in these expressions must be large enough to ensure that the oscillations of e^i^*^β^* in Eqs. (7d,e) are accurately sampled. According to Eq. (7a) and the quarter-wave criterion this will be achieved if the pathlength difference Δ*PQ* between successive summation elements is less than *λ*/4, or Δ*β* ≤ π/2. Differentiating Eq. (7a) with respect to *v* gives

$$\frac{kw}{N}=\Delta v=\frac{\beta \;\Delta \beta }{v}<\frac{\pi \;\beta }{2v},\;N>\frac{2v\;kw}{\pi \;\beta },$$ (8e)

which can now be used as follows to estimate the required value of *N*. In the immediate vicinity of the screen (*z* → 0 we have *β* ~ v, so that *N* > 2*w*/*λ* = 100 when *w* = 50*λ*. In the Fresnel limit (*z* >> *λ*) one finds *N* > 2 *v*_max_*w*/π*z* where *v*_max_ is the largest value of *v* used in the computations, and therefore *N* > 6*w*^2^/*λz* = 150 when *w* = 50*λ*, *z* = 100*λ* and the farthest point of observation is located one aperture halfwidth beyond the shadow boundary (*v*_max_ = 1.5 *kw*). It should be noted that the corresponding values of *N* can be much larger for wider apertures.

##### 3.3.2.2 Numerical Examples

###### 1. Forward and Reverse Axial Irradiances

Equations (7f,g) can be solved in closed form for the special case of axial points of observation, where *x* = 0, *χ* = 0 and $\beta =k\sqrt{\xi^{2}+z^{2}}$ and therefore

$$\begin{array}{l} u_{\mathrm{RS}}^{\left(p\right)}\left(0,z\right)=-ik^{2}\int_{0}^{kw}d\xi \xi \frac{\exp\left(i\beta \right)}{\beta }\\ \;=-\int_{kz}^{kW}d\;t\exp\left(t\right)=\exp\left(i\;kz\right)-\exp\left(i\;kW\right),\\ t=i\beta ,\;W=\sqrt{w^{2}+z^{2}}. \end{array}$$ (9a)

Hence one finds, using Eqs. (7e) and (6e),

$$\begin{array}{l} u_{\mathrm{RS}}^{\left(s\right)}\left(0,z\right)=\exp\left(ikz\right)-\frac{z\exp\left(ikW\right)}{W},\\ u_{K}\left(0,z\right)=\exp\left(ikz\right)-\frac{1}{2}\exp\left(ikW\right)\left(1-\frac{z}{W}\right),\\ {\hat{u}}_{K}\left(0,z\right)-\frac{1}{2}\exp\left(ikW\right)\left(1+\frac{z}{W}\right), \end{array}$$ (9b)

which can now be substituted into Eqs. (6i, j) and then leads to the following expressions for the forward and reverse irradiances along the *z*-axis of Fig. 2,

$$\begin{array}{l} E\left(0,z\right)=E_{\mathrm{geom}}{\{1+\frac{1}{4}\left(1-\frac{\left|z\right|}{W}\right)}^{2}\\ -\cos\left[k\left(z-W\right)\right]\left(1-\frac{\left|z\right|}{W}\right)\},\\ \hat{E}\left(0,z\right)=\frac{1}{4}E_{\mathrm{geom}}{\left(1-\frac{\left|z\right|}{W}\right)}^{2}. \end{array}$$ (9c)

**Fig. 3:**
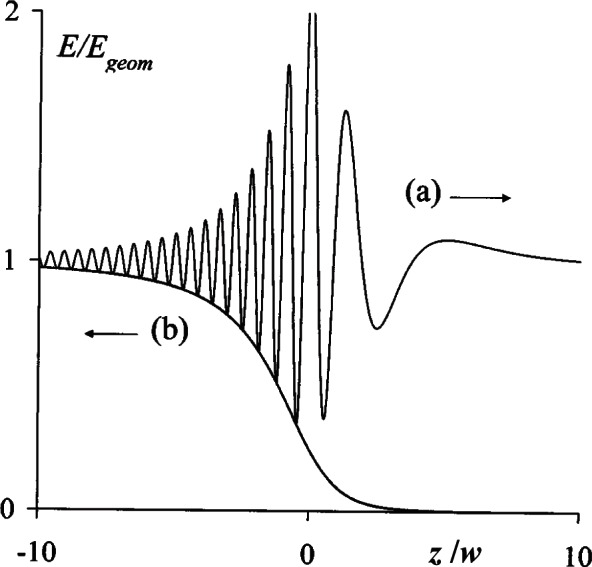
Forward and reverse axial irradiances, (a) *E*_K_ and (b) *Ê*_K_, on opposite sides of a circular aperture of radius *w* = 5*λ*.

These results are valid for arbitrary values of *z* and are plotted in Fig. 3, where the upper curve represents the forward axial irradiance *E*(0, *z*) computed from the first Eq. (9c) for *w* = 5*λ* and the lower curve represents the corresponding reverse irradiance *Ê*(0, *z*). Both quantities are continuously differentiable on crossing the aperture plane, the forward irradiance (a) being equal to the geometrical irradiance *E*_geom_ in the negative Fresnel limit − *z* >>*λ*, oscillating rapidly in the vicinity of the aperture, and tapering off beyond it. The reverse axial irradiance (b) is seemingly diverging from a virtual source point beyond *z* = − 10*λ*, exhibits no oscillations, and its magnitude is equal to the lower envelope of the forward irradiance curve.

###### 2. Near-Field Diffraction Patterns

**Fig. 4:**
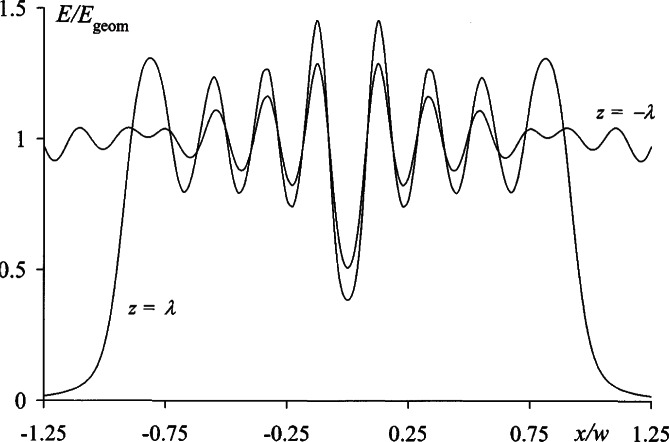
Forward irradiance profiles *E*_K_ at distances *z* = ±*λ* from a circular aperture of radius *w* = 5*λ*.

**Fig. 5:**
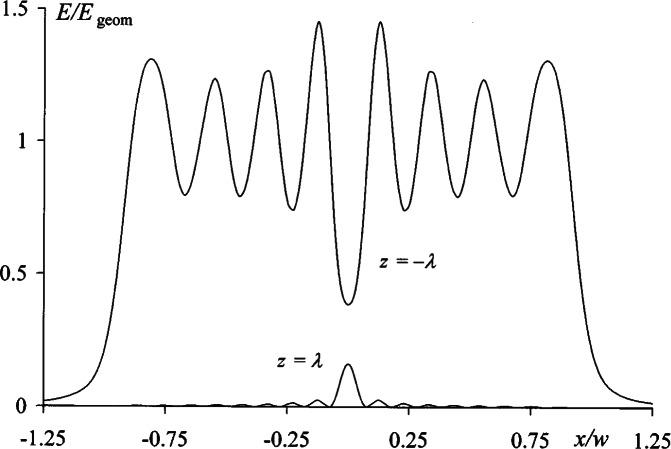
Reverse irradiance profiles *Ê*_K_ at distances *z* = ±*λ* from a circular aperture of radius *w* = 5*λ*.

The application of Eqs. (8a–c) for the computation of diffraction patterns is straightforward, and in this work standard spreadsheet software was used to obtain numerical results. As an example, Fig. 4 shows the near-field irradiance profiles (6i) on the opposite sides (*z* = ±*λ*) of the previously considered circular aperture of radius *w* = 5*λ*. The resemblance of the central portions of these curves is remarkable and is attributable to the basic premise adopted in Sec. 3.2, where St. Venant’s hypothesis was replaced with the assumption that diffraction is a continuous field phenomenon that occurs on both sides of the screen. The corresponding reverse profiles defined by Eq. (6j) are shown in Fig. 5, illustrating the onset of a reverse flow of energy on the positive side of the screen (*z* = *λ*) as well as the fact that the reflected diffraction pattern on the negative side (*z* = −*λ*) is simply a mirror image of the transmitted pattern in Fig. 4.

###### 3. Aperture Field and Transmission Coefficients

For radiometric applications it is important to know the transmission coefficient of an aperture, defined as *τ* = Φ_total_ / Φ_geom_ where Φ_total_ is the total radiant flux transmitted into the half space *z* > 0 and Φ_geom_ is the geometrical flux incident upon it in the absence of diffraction. Thus, for a circular aperture as discussed in this Section,

$$\begin{array}{l} \tau =\frac{1}{AE_{\mathrm{geom}}}\int_{A}\mathrm{dQ}\;E\left(Q\right)=\frac{2}{w^{2}}\int_{0}^{w}dx\;x|u_{K}\left(x,0\right){|}^{2}\\ \;=\frac{2}{N^{2}}\sum_{m=1}^{N}\left(m-\frac{1}{2}\right)|u_{K}\left(m,0\right){|}^{2}, \end{array}$$ (10a)

where *A* = π*w*^2^ is the aperture area, dQ = 2π*x* d*x* is the circular area element, and *u*_K_ (*m*, *z*) is given by Eq. (8a). The practical use of Eq. (10a) is tedious because it requires consecutive numerical integrations and also poses computational problems arising from the singularities of *u*_K_(*x*, *z*) in the limit *z* → 0. The most troublesome singularities, due to the terms in 1/*β*^2^ and 1/*β*^3^ in Eq. (7f), can be avoided altogether by invoking the second Eq. (5e) so that

$$u_{K}\left(m,0\right)\approx \frac{1}{2}\left[1+u_{\mathrm{RS}}^{\left(p\right)}\left(m,z_{\min}\right)\right],$$ (10b)

where $u_{\mathrm{RS}}^{\left(p\right)}$ is singular in 1/*β*, only, and *z*_min_ is very small. Trial computations indicated that values of *z*_min_ as small as 0.0003*λ* could be used without difficulty and that the limiting value of *τ* defined by Eq. (10a) was reached at the 0.1 % level for *z*_min_ < 0.0003*λ*. Accordingly, the numerical result presented in the following were computed for *z*_min_ = 0.001*λ*. As expected, the aperture irradiance distributions |*u*_K_(*x*, 0)|^2^ obtained from Eq. (10b) were similar to an average of the two curves in Fig. 5. They were everywhere finite and continuous and bore no similarity to the aperture distributions presumed by Rayleigh and Bouwkamp [6,10], Levine and Schwinger [11] or Wolf and Marchand [15].

**Fig. 6:**
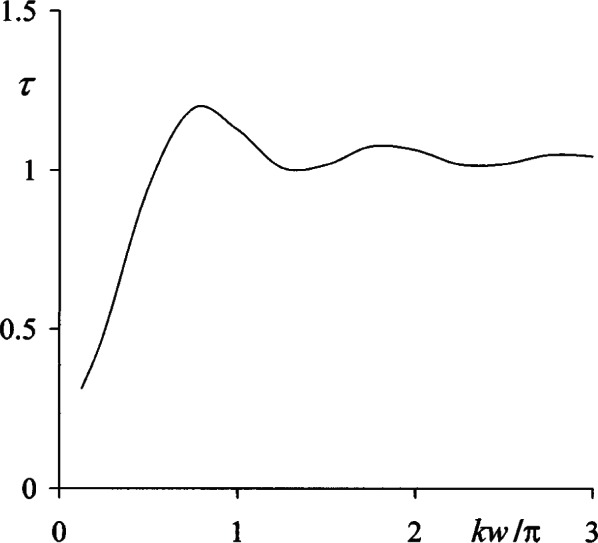
Transmission coefficient *τ* of circular apertures vs aperture radius *w*.

Figure 6 shows the dependence of the transmission coefficient (10a) on aperture size for the range 0 < *kw* < 3π, and here it is seen that *τ* exhibits a damped oscillatory behavior and quickly approaches the limit, *τ* → 1 as *kw* → ∞. It was estimated that this limit is reached within less than 1 % when *w* = 5*λ*.

#### 3.3.3 Apertures Bounded by Straight Edges

##### 3.3.3.1 Algorithms

**Fig. 7:**
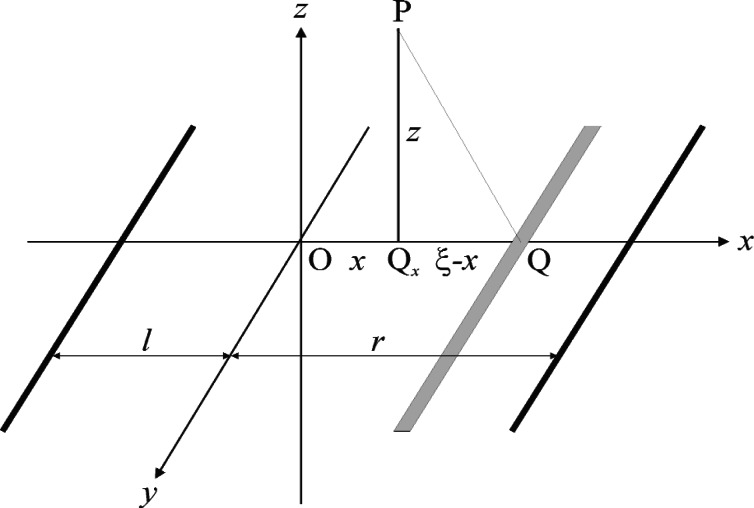
Notation used for slits.

Consider a plane aperture of width (*l* + *r*), bounded by parallel straight edges as indicated in Fig. 7. The corresponding diffraction pattern will consist of straight bands which are parallel to the edges, and thus it will again be sufficient to compute its variation along the *x*-axis. For a given point of observation P = (*x*, 0, *z*) and arbitrary aperture points Q = (*ξ*, *η*, 0), Eqs. (5a,b) can now be expressed as follows [12],

$$\begin{array}{l} u_{\mathrm{RS}}^{\left(p\right)}\left(x,z\right)=-\frac{ik}{2\pi }\int_{-l}^{r}d\xi \int_{-\infty }^{\infty }\frac{d\eta \exp[ik\sqrt{{\left(\xi -x\right)}^{2}+\eta^{2}+z^{2}}}{\sqrt{{\left(\xi -x\right)}^{2}+\eta^{2}+z^{2}}}\\ \;=\frac{1}{2}\int_{-k\left(l+x\right)}^{k\left(r-x\right)}dvH_{0}^{\left(1\right)}\left(\beta \right),\\ u_{\mathrm{RS}}^{\left(s\right)}\left(x,z\right)=\frac{ikz}{2}\int_{-k\left(l+x\right)}^{k\left(r-x\right)}\frac{dvH_{1}^{\left(1\right)}\left(\beta \right)}{\beta }, \end{array}$$ (11a)

where *z* > 0, $H_{0}^{\left(n\right)}=J_{n}+{\mathrm{iY}}_{n}$ denotes a Hankel function, J*_n_* and Y*_n_* are Bessel functions, and *v* as well as *β* are the same as in Eq. (7a). It follows at once that the forward and reverse wave functions defined in Sec. 3.2 are given by

$$\begin{array}{l} u_{K}\left(x,z\right)=\frac{1}{2}\left[u_{\mathrm{RS}}^{\left(p\right)}\left(x,z\right)+u_{\mathrm{RS}}^{\left(s\right)}\left(x,z\right)\right]\\ \;=\int_{-k\left(l+x\right)}^{k\left(r-x\right)}dv\;S\left(\beta \right),\;z>0,\\ S\left(\beta \right)=\frac{1}{4}\left[J_{0}\left(\beta \right)-\frac{kz}{\beta }Y_{1}(\beta \right]\\ \;+\frac{i}{4}\left[Y_{0}\left(\beta \right)+\frac{kz}{\beta }J_{1}(\beta \right], \end{array}$$ (11b)

$$\begin{array}{l} {\hat{u}}_{K}\left(x,z\right)=\frac{1}{2}\left[u_{\mathrm{RS}}^{\left(p\right)}\left(x,z\right)-u_{\mathrm{RS}}^{\left(s\right)}\left(x,z\right)\right]\\ \;=\int_{-k\left(l+x\right)}^{k\left(r-x\right)}dv\;\hat{S}\left(\beta \right),\;z>0,\\ \hat{S}\left(\beta \right)=\frac{1}{4}\left[J_{0}\left(\beta \right)+\frac{kz}{\beta }Y_{1}(\beta \right]\\ \;+\frac{i}{4}\left[Y_{0}\left(\beta \right)-\frac{kz}{\beta }J_{1}(\beta \right]. \end{array}$$ (11c)

To evaluate these expressions by numerical integration define, in analogy to the definitions that precede Eqs. (8a)*l*+*r* = 2*w*, Δ*v* = *kw*/*N*, *L* = 2*kl*/Δ*v*, *R* = 2*kr*/Δ*v*, *v* = *n*Δ*v*, *kx* = *m*Δ*v*. Therefore,

$$u_{K}\left(x,z\right)=u_{K}\left(m,z\right)=\frac{kw}{N}\sum_{n=-\left(L+m\right)}^{R-m}S\left(\beta_{n}\right),\;z>0,$$ (12a)

$${\hat{u}}_{K}\left(x,z\right)={\hat{u}}_{K}\left(m,z\right)=\frac{kw}{N}\sum_{n=-\left(L+m\right)}^{R-m}\hat{S}\left(\beta_{n}\right),\;z>0,$$ (12b)

where, as before, $\beta_{n}=\sqrt{\left[{\left(n-\frac{1}{2}\right)}^{2}{\left(kw\right)}^{2}/N^{2}\right]+{\left(kz\right)}^{2}}$ and the choice of *N* is governed by the same considerations as in Sec. 3.3.2.1. As *β_n_* is independent of *m* it follows at once that, if *u*_K_(*m*, *z*) and û_K_(*m*, *z*) are known and *m* is replaced by *m* ± 1, the new values will be

$$\begin{array}{l} u_{K}\left(m\pm 1,z\right)=u_{K}\left(m,z\right)\mp \frac{kw}{N}\left[S\left(\beta_{-L+m\pm 1}\right)-S\left(\beta_{R-m\pm 1}\right)\right],\\ z>0, \end{array}$$ (12c)

$$\begin{array}{l} {\hat{u}}_{K}\left(m\pm 1,z\right)={\hat{u}}_{K}\left(m,z\right)\mp \frac{kw}{N}\left[\hat{S}\left(\beta_{-L+m\pm 1}\right)-\hat{S}\left(\beta_{R-m\pm 1}\right)\right],\\ z>0, \end{array}$$ (12d)

which illustrates in a very instructive manner how the diffraction pattern changes when the point of observation is moved so that new portions of the wavefront are covered and uncovered by the aperture edges. The recursion formulae (12c,d) are convenient for practical applications as they allow the computation of successive values without performing the summations of Eqs. (12a,b) for every point of observation.

The results obtained for the diffraction profiles of slits are similar to those presented in Sec. 3.3.2.2 for circular apertures. They were included in Ref. [12] and are omitted here.

### 3.4 Half-Plane Results and Comparison With Sommerfeld’s Theory

The aperture depicted in Fig. 7 is reduced to an infinitesimally thin half plane defined by *x* > 0, *z* = 0, by letting *L* = − ∞, *R* = 0. Accordingly, Eqs. (12a–d) are now replaced by

$$\begin{array}{l} u_{K}\left(x,z\right)=\int_{-\infty }^{-kx}dv\;S\left(\beta \right)=u_{K}\left(m,z\right)=\Delta v\sum_{n=-\infty }^{-m}S\left(\beta_{n}\right),\\ u_{K}\left(m\pm 1,z\right)=u_{K}\left(m,z\right)\mp \Delta v\;S\left(\beta_{m\pm 1}\right),\;z>0, \end{array}$$ (13a)

$$\begin{array}{l} {\hat{u}}_{K}\left(x,z\right)=\int_{-\infty }^{-kx}dv\;\hat{S}\left(\beta \right)={\hat{u}}_{K}\left(m,z\right)=\Delta v\sum_{n=-\infty }^{-m}\hat{S}\left(\beta_{n}\right),\\ {\hat{u}}_{K}\left(m\pm 1,z\right)={\hat{u}}_{K}\left(m,z\right)\mp \Delta v\;\hat{S}\left(\beta_{m\pm 1}\right),\;z>0, \end{array}$$ (13b)

where it should be noted that the last terms on the right-hand sides of Eqs. (12c,d) are now absent because there is no right aperture edge. Here, as above, *kx* = *m*Δ*v*, *k*(*ξ* − *x*) = *n*Δ*v*, *S*(*β_n_*) and *Ŝ*(*β_n_*), being the same as in (11b,c), and $\beta_{n}=\sqrt{{\left(n-\frac{1}{2}\right)}^{2}\Delta v^{2}+{\left(kz\right)}^{2}}$. The summation elements Δ*v* must again satisfy the quarter-wave criterion, so that the phase difference between successive summation elements, Δ*β* = *β_n +_*1 − *β_n_*, must not exceed π/2. This phase difference has a maximum value, (Δ*β*)_max_ = Δ*v* when *z* = 0, and hence it follows that the quarter-wave condition will always be satisfied when Δ*v* < π/2. As the choice of Δ*v* also determines the step size of the recursions (13a,b) the value chosen in this work was Δ*v* = π/5, yielding equidistant values of *u*_K_(*x*, *z*) and *û*_K_(*x*, *z*) spaced by Δ*x* = π/5*k* = 0.1*λ*. The starting values, *u*_K_(0, *z*) = 0.5 and *û*_K_(0, *z*) = 0, were obtained by performing the summations in Eqs. (13a,b) for *m* = 0.

The corresponding expressions according to Sommerfeld’s rigorous theory [7, 9, 12] for the border case of a normally incident geometric field are

$$u_{S}^{\left(p,s\right)}\left(x,z\right)=u_{S}\left(x,z\right)\pm {\hat{u}}_{S}\left(x,z\right),\;z\neq 0,$$ (14a)

$$\begin{array}{c} u_{S}\left(x,z\right)=\exp\left(ikz\right)\;V\left(\rho \right),\;\rho =\sqrt{2/\lambda }[-\mathrm{sign}\left(z\right)\\ \sqrt{r+x}+\sqrt{r-x}],\;z\neq 0, \end{array}$$ (14b)

$$\begin{array}{c} {\hat{u}}_{S}\left(x,z\right)=\exp\left(\text{-}ikz\right)\;V\left(\hat{\rho }\right),\;\hat{\rho }=\sqrt{2/\lambda }[-\mathrm{sign}\left(z\right)\\ \sqrt{r+x}-\sqrt{r-x}],\;z\neq 0, \end{array}$$ (14c)

$$\begin{array}{c} V\left(\rho \right)=\frac{\exp\left(\text{-}i\pi /4\right)}{\sqrt{2}}\int_{-\infty }^{\rho }d\tau \exp\left(i\pi \tau^{2}/4\right)=\frac{1}{2}[1+C\left(\rho \right)\\ +S\left(\rho \right)]-\frac{i}{2}\left[C\left(\rho \right)-S\left(\rho \right)\right], \end{array}$$ (14d)

where $u_{S}^{\left(p,s\right)}\left(x,z\right)$ satisfy the wave equation and the respective boundary conditions $\partial u_{S}^{\left(p\right)}/\partial z=0$ and $u_{S}^{\left(s\right)}=0$, *u*_S_ and *û*_S_ are the forward and reverse wave functions, *V*(*ρ*) is the complex Fresnel integral the form used by Sommerfeld,^6^ while C(*ρ*) and S(*ρ*) are the standard Fresnel cosine and sine integrals.

**Fig. 8:**
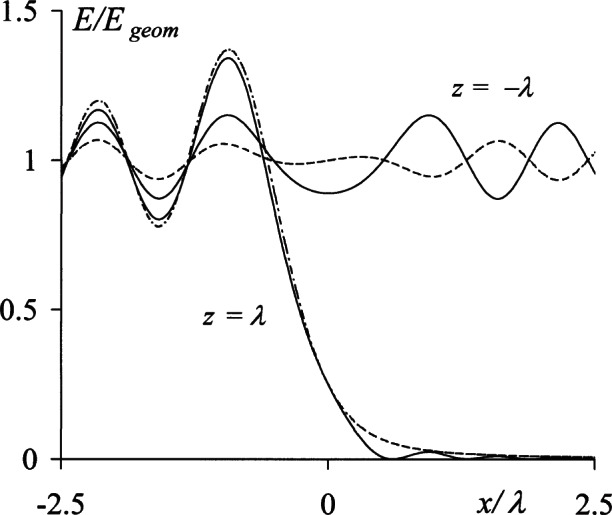
Forward irradiance profiles *E*_K_ at distances *z* = ±λ from a half plane according to Eqs. (13a) (-----) and (14b) (––––).

During the course of this work, the above sets of equations were used for numerous computations intended to quantify the residual differences between them. The results obtained showed consistently that these differences were insignificant even in the immediate vicinity of the diffracting edge. As an example, Fig. 8 shows the respective forward irradiance distributions, | *u*_K_ |^2^ and | *u*_S_ |^2^, at the distances *z* = ± *λ* from the half plane. The similarity of the curves is unmistakable, and further computations showed that the same quantities differ by less than ± 0.01 for *z* = ± 10*λ*. This demonstrates the rigor of the equations derived in Sec. 3.2 for the specific case of half-plane diffraction, and it may be inferred that the corresponding expressions for circular apertures and slits are equally as rigorous.

## 4. A Note on Electromagnetic Diffraction Theories

The thoughts presented in this paper arose from a realization that, at some time in the mid 1900’s, the theory of diffraction had reached an impasse. Although it should have been obvious that the inability to account for polarization phenomena is an intrinsic feature of the scalar theory of light, and although there was no experimental evidence that this is a practical problem in the context of Fresnel’s theory,^7^ attempts were made to modify the theory so that, in a manner of speaking, polarization is introduced through the back door and the scalar theory assumes a pseudo-vectorial character. These modifications were justified as follows [e.g., 10, 16].

For infinite plane screens and plane-parallel incident light, the Helmholtz integral theorem [5] has two mutually independent solutions, *u*^(^*^p^*^)^ and *u*^(^*^s^*^)^, which include but are not limited to the Rayleigh-Sommerfeld and Sommerfeld solutions $u_{\mathrm{RS}}^{\left(p,s\right)}$ and $u_{S}^{\left(p,s\right)}$, discussed in Sec. 3 and obey the same boundary conditions as those pertaining to the reflection of polarized light at a perfectly conducting metallic reflector. Hence it is surmised, without further proof, that these solutions can be regarded as the components of mutually independent electromagnetic fields given by $H^{\left(p\right)}=\sqrt{\varepsilon /\mu }\left(0,u^{\left(p\right)},0\right)$, $i\;kE^{\left(p\right)}=-\sqrt{\mu /\varepsilon }\;\mathrm{curl}\;H^{\left(p\right)}$ and $E^{\left(s\right)}=\sqrt{\mu /\varepsilon }\left(0,u^{\left(s\right)},0\right)$, $i\;kH^{\left(s\right)}=\sqrt{\varepsilon /\mu }\;\mathrm{curl}\;E^{\left(s\right)}$, where SI units are used and $\sqrt{\mu /\varepsilon }$ is the wave impedance of free space.

It is easy to show that these assumptions are fallacious and can lead to contradictory or dubious results. For example:

According to the above, unpolarized light incident on a diffracting screen is transmitted as partially polarized light having a polarization ratio given by Π = |*u*^(^*^p^*^)^|^2^/|*u*^(^*^s^*^)^|^2^ and ordinarily this ratio would not be unity in the near zone. However, in the mid zone *u*^(^*^p^*^)^ and *u*^(^*^s^*^)^ are expected to be same so that Π = 1. This is absurd, because the polarization of light cannot change while propagating in free space.
The curl operators in Maxwell’s equations are known to introduce singularities into the “electromagnetic” theory, even when the underlying wave functions *u*^(^*^p^*^)^ and *u*^(^*^s^*^)^ are everywhere finite and continuous. These singularities have been interpreted as evanescent edge waves emitted by the diffracting edge, as originally presumed by Thomas Young but soon refuted by Fresnel.^8^ The notion of edge waves has persisted as a means to explain singularities encountered in diffraction calculations [18], whereas a thorough analysis might have revealed that these singularities are artifacts of mathematical errors or illogical assumptions.

The reason for inconsistencies of this kind is that the conditions *u* ^(^*^p^*^)^ = 0 and ∂*u*^(^*^s^*^)^/∂*z* = 0 are necessary and sufficient to satisfy Helmholtz’ theorem, but insufficient to transform the scalar theory of light into a viable tool for explaining electromagnetic phenomena.

## Figures and Tables

**Table 1:** Smallest permissible source-aperture distance *r*_0,min_ defined by Eq. (3a) for selected aperture sizes 2*w* (left column) and source sizes 2*s* (top row), *λ* = 1 μm

2*w* / 2*s*	0.01 mm	0.1 mm	1 mm	10 mm
0.1 mm	6 mm	20 mm	605 mm	50.1 m
1 mm	510 mm	605 mm	2 m	60.5 m
10 mm	50 m	51 m	60.5 m	200 m

## Footnotes

1 This inequality for *r* 0 follows from Eq. (4a) for *s* = 0.

2 Jean Claude Saint-Venant (1797–1866) taught mathematics at the École des Ponts et Chaussées in Paris, where Fresnel studied engineering between 1806 and 1809.

3 At the time of this collaboration with Fresnel, Francois Jean Dominique Arago (1786–1853) was Secretary of the Paris Observatory. For a brief time in the Spring of 1848 he served as the Head of the Second French Republic.

4 Equation (4a) is more stringent than a well-known coherence criterion attributed to Verdet (1869) which specifies different minimum distances, *r*_0,min_ = 8*sw*/*λ.* These are also quite large for large apertures and sources.

5 Helmholtz’ theorem was formulated as a mathematical theory of organ pipes, and thus also implies the assumption of longitudinal waves.

6 Different notations have been used elsewhere. For example, in Ref. [17] therse relationships were expressed of the function $F\left(-\sqrt{\pi /2}\rho \right)=\exp\left(i\pi /4\right)\sqrt{\pi }V\left(\rho \right)$.

7 This may be due to the paraxial approximations made in Fresnel’s theory. The existence of polarization effects at large angles is, of course, evidenced by the properties of diffraction gratings.

8 It appears that Young did not publish this hypothesis and that Fresnel was the first to mention it.
